# Stakeholders' perceptions of child and adolescent mental health services in a South African district: a qualitative study

**DOI:** 10.1186/s13033-020-00406-2

**Published:** 2020-10-02

**Authors:** Gbotemi Bukola Babatunde, André Janse van Rensburg, Arvin Bhana, Inge Petersen

**Affiliations:** Centre for Rural Health, School of Nursing & Public Health, University of KwaZulu-Natal, Durban, South Africa

**Keywords:** Mental health services, Child and adolescent mental health, District child and adolescent mental health plan, District mental health services, Intersectoral collaboration

## Abstract

**Background:**

In order to develop a district child and adolescent mental health (CAMH) plan, it is vital to engage with a range of stakeholders involved in providing CAMH services, given the complexities associated with delivering such services. Hence this study sought to explore multisectoral dynamics in providing CAMH care in one resource-constrained South African district as a case study, towards informing the development of a model for district mental health plan and generating lessons for mental health systems strengthening to support CAMH services using the Health Systems Dynamics (HSD) framework. HSD provides a suitable structure for analysing interactions between different elements within the health system and other sectors.

**Methods:**

Purposive sampling of 60 key informants was conducted to obtain an in-depth understanding of various stakeholders' experiences and perceptions of the available CAMH services in the district. The participants include stakeholders from the Departments of Health (DoH), Basic Education (DBE), community-based/non-governmental organizations and caregivers of children receiving CAMH care. The data was categorized according to the elements of the HSD framework.

**Results:**

The HSD framework helped in identifying the components of the health systems that are necessary for CAMH service delivery. At a district level, the shortage of human resources, un-coordinated CAMH management system, lack of intersectoral collaboration and the low priority given to the CAMH system negatively impacts on the service providers' experiences of providing CAMH services. Services users' experiences of access to available CAMH services was negatively impacted by financial restrictions, low mental health literacy and stigmatization. Nevertheless, the study participants perceived the available CAMH specialists to be competent and dedicated to delivering quality services but will benefit from systems strengthening initiatives that can expand the workforce and equip non-specialists with the required skills, resources and adequate coordination.

**Conclusions:**

The need to develop the capacity of all the involved stakeholders in relation to CAMH services was imperative in the district. The need to create a mental health outreach team and equip teachers and caregivers with skills required to promote mental wellbeing, promptly identify CAMH conditions, refer appropriately and adhere to a management regimen was emphasized.

## Background

There has been increased attention paid to improving child and adolescent mental health (CAMH) services in different countries around the globe [29, 47]. Several policy documents have helped to spur this [49–51], notably the World Health Organization's (WHO) policy framework for *Child and Adolescent Mental Health Policies and Plans* [49]. However, the paucity of specific national CAMH policies and national implementation guidelines, poor intersectoral collaboration and the shortage of CAMH resources still hinder the provision of optimal child and adolescent mental health services in many countries [25].

The burden of CAMH has been well-described, especially in LMICs [17, 37]. Barriers to CAMH service provision in LMICs will undoubtedly be aggravated by the COVID-19 pandemic, an event that will substantially test the resilience and responsiveness of district health systems. It has already been noted that the pandemic will add to the current CAMH burden, and a strong system of governance, service provision and financing will be vital to ensure the well-being of children and adolescents [19].

Two considerations have especially been part of strategies to reform CAMH services, namely task-sharing and intersectoral working. While CAMH services have historically been framed to be the sole responsibility of specialists, some recent studies have revealed the possibility and significance of integrating CAMH services into primary health care (PHC) through the task-sharing approach [25, 30, 35, 47]. Notably, the Mental Health GAP project (mhGAP) [51] includes guidelines for the management of several CAMH conditions at PHC level within a task-sharing approach. In terms of intersectoral working, CAMH has historically been under the stewardship of the health sector. An intersectoral approach that involves the collaboration of other sectors such as education, social development and juvenile justice is required to achieve an effective CAMH system of care [10, 11].

While these considerations have been central to South Africa's health policy landscape, the country lacks a well-articulated CAMH strategy which is required to achieve a functional CAMH collaborative system at a district level [27, 33]. In the development of such a strategy, there is a need to involve a wide variety of stakeholders across multiple sectors, including caregivers, teachers, community and spiritual leaders [26]. Haine-Schlagel et al. [22], emphasized that engaging various stakeholders was critical to achieving an effective CAMH service delivery. These multiple stakeholders, particularly teachers and caregivers (parents, grandparents, foster parents and other family members), are perceived to be active gatekeepers to CAMH care, given their vital role in identifying and seeking help for children and adolescents with mental (behavioural, emotional, social and developmental) disorders.

Despite the inclusion of CAMH in core national documents like the Policy Guidelines on Child and Adolescent Mental Health [14] and the National Mental Health Policy Framework and Strategic Plan 2013–2020 [16], within the ideals of integrated, collaborative care (including task-sharing and intersectoral working, little to no guidance exists for provincial and district governments to translate national guidelines into operational tools for district governance of CAMH services. Considering this, the study aimed to explore multisectoral dynamics in providing CAMH care in one resource-constrained South African district as a case study, towards informing the development of a model for district mental health plan and generating lessons for mental health systems strengthening to support CAMH services.

The study was guided by the Health Service Delivery (HSD) framework which describes Health service delivery as a process by which policies, services providers and infrastructures are organized to achieve the goal of the health system which is to provide easily accessible and quality healthcare services [46]. The framework consists of ten elements, they include, (1) goals and outcomes, (2) values and principles, (3) service delivery, (4) the population, (5) the context, (6) leadership and governance, (7) finances, (8) human resources, (9) infrastructure and supplies, (10) knowledge and information. The premise of the HSD framework is that the health system is an open system which is often shaped and influenced by different societal factors. It describes health service delivery as a process by which policies, services providers and infrastructures are organized to achieve the goal of the health system which is to provide easily accessible and quality healthcare services. Moreover, resources such as budget allocation, human resources, infrastructure and supplies, knowledge and information are fundamental to achieving a viable healthcare system for the populace. The population (service users) are described as major players within the health system. The authors emphasized that they are not mere patients but also citizens having rights to access quality healthcare. Governance, as described by the HSD framework, entails policy guidance, coordination of the different stakeholders and activities at different levels of care and effective distribution of resources to ensure equity and accountability [46].

## Methods

### Research design

An instrumental case study which is used to obtain an in-depth understanding of specific issues was conducted with the Amajuba District Municipality as the unit of analysis [42] to explore the experiences of providing and accessing CAMH services in the district. Employing a phenomenological qualitative approach using semi-structured interviews, the design allowed for the generation of in-depth information about lived experiences from multiple stakeholder perspectives [38].

### Study setting

The study was conducted in the Amajuba District Municipality, in the north-west region of the KwaZulu-Natal province of South Africa. The district which covers 6911 km^2^ with a population estimate of about 442,266, is made up of 3 sub-districts and comprises rural and peri-urban communities [1, 20]. Amajuba has been identified as a resource-constrained district as it has limited numbers of health professionals, including mental health specialists to provide adequate health care services for the populace [20]. The bulk of the district's CAMH service capacity is situated in its three provincial hospitals. The district was a site for government piloting of the National Health Insurance programme- a government-driven initiative aimed to unify South Africa's two-tiered health system by establishing a centralised funding mechanism in order to achieve universal health coverage [15]. As part of its pilot site status, the district had limited school mental health services as part of the Integrated School Health Programme, an extension of the revitalisation of PHC, that includes teams of health care workers (HCWs) visiting schools to conduct basic screening and referral services [3, 31].

### Participant selection and characteristics

Research participants were purposively identified according to their positions in the departments of health, social development and education. Snowball selection was applied, leading to the identification and participation of 60 key role players involved in providing mental health care to children and adolescents in the district. Participants included managers and mental health professionals from the Department of Health, managers, educators and mental health support workers from the Department of Basic Education, non-governmental service representatives, as well as caregivers of children and adolescents living with mental health challenges. A list of CAMH cases and conditions identified in the district over 12 months have been published elsewhere [6]. These conditions included autism spectrum disorder, attention-deficit/hyperactivity disorder (ADHD), different forms of intellectual disability, depression, schizophrenia, bipolar affective disorders, mood disorder, anxiety, conduct disorder, mental and behavioral disorders tied to substance abuse. A full list of participants and the characteristics of children whose caregivers were included in this study are presented in Tables 1 and 2.

**Table 1:** Participants’ categories

Participant category	Frequency	Location
Department of Health (DOH) (Health professionals)		
District mental health coordinator (DMHC)	1	DoH district office
Psychiatrist	1	Provincial hospital A
Medical officer	2	Provincial hospital A and C
Psychologists	2	Provincial hospital A and B
Psychiatric nurses	6	Provincial hospital A
School health nurses	3	Provincial hospital A
Pharmacists	2	Provincial hospital A
Clinic operational manager	1	PHC clinic
Psychiatric outpatient operational manager	1	Provincial hospital A
Occupational therapists	2	Provincial hospital A and B
Social workers	2	Provincial hospital A
Total number of DOH participants	23	
Department of Basic Education (DBE)		
Learning support agents in schools (LSA)	3	Primary and high schools
Educators	6	Primary and high schools
School principals	3	Primary and high schools
School-based occupational therapist	1	Special school
Special school chief director	1	DBE district office
Total number of DBE participants	14	
Caregivers		
Mothers	10	Provincial hospital A and B
Father	1	Provincial hospital B
Grandmothers	3	Provincial hospital A
Other relatives	6	Provincial hospital A
Total number of caregivers	20	
Non-governmental organizations (NGOs)		
District program coordinator, South African National Council on Alcoholism and Drug Dependence (SANCA)	1	Newcastle
CAMH community volunteers	2	Madadeni
Total number of NGO participants	3	
Total of participants	60

**Table 2:** Children’s characteristics and CAMH conditions

Gender	Condition	Ages	Frequency
Males	Autism spectrum disorder	5, 6, 7, 16, 17	5
Females	Autism spectrum disorder	6, 6, 7	3
Males	Attention deficit hyperactivity disorder (ADHD)	6, 7, 9	3
Females	ADHD	9	1
Males	ADHD with epilepsy	12	1
Female	ADHD with epilepsy	8	1
Males	Intellectual disability	12, 10, 15	3
Females	Intellectual disability	9, 15	2
Males	Mental and behavioral disorder due to substance abuse	16, 17	2
	Total		21

### Data gathering

Data gathering for this study took place from February to March 2019. Semi-structured interviews were used, allowing for the use of probes and follow-up questions to steer the discussion while allowing for the generation of in-depth subjective information [4, 13]. The interview guide was informed by the findings of an initial review of literature on the barrier and facilitators of CAMH services in low- and -middle-income countries [5] and the HSD framework. The interview guide covered a range of questions that explored the roles played by each stakeholder in relation to CAMH services, their perceptions, and experiences of child and adolescent mental health; experiences of accessing and providing CAMH services, and suggested pathways for systems improvement. All the stakeholders included in this study were either physically visited in their offices or contacted via e-mail, text messages, and telephonically to inform them and solicit their participation in the study. The majority of the stakeholders responded positively, and interview dates and time were secured.

The operational manager at the Madadeni hospital psychiatric out-patient department and the clinical psychologist at the Newcastle hospital assisted with identifying caregivers and introduced them to the researchers. The caregivers were then informed about the study during clinic days and twenty caregivers consented to participate in the study. Interviews were conducted in English and isiZulu, depending on interviewee preference. The primary researcher (GBB), a doctoral student, conducted the English interviews while the IsiZulu interviewers were conducted by a trained research assistant with a bachelor's degree, who is proficient with the use of both isiZulu and English language. The research assistant is also a resident of the community, and this facilitated easy rapport with the stakeholders. The interviews were audio-recorded, transcribed verbatim, translated, and back-translated where required.

### Data analysis

Transcribed data were analysed using Gale et al.’s [18] framework method, a summary process for managing and analysing qualitative data, which produces a series of themed matrices [44]. Accordingly, six steps were followed: (1) transcription, (2) familiarisation, (3) deductive organisation of codes based on the elements of the HSD framework, (4) inductive coding of sub-themes under the HSD coding framework, (5) reviewing data extract and charting (6) mapping and interpretation of data [18, 40]. Using these interconnected steps enabled the researchers to sort, scrutinise, categorise and chart the themes and associated sub-themes that emerged from the data set [8, 48]. The categories were reviewed to identify existing connections and differences between the themes from the different groups of stakeholders [45]. The excel software package (2019) was used in creating framework matrices and coding the entire data set. The accuracy of transcripts was checked against original recordings, and the two researchers (GBB and AV) who conducted the analysis compared results at regular time points to harmonise the content of themes derived from raw data. Also, the classification was discussed iteratively between the researchers, with input from study supervisors (AB and IP). To further ensure trustworthiness, the data set was thoroughly read through to confirm that the data was meaningfully clustered under the appropriate themes. Furthermore, member checking took place to validate and verify the results. Gatekeeper permission was obtained from the relevant government departments, and ethics approval was provided by the Biomedical Research Ethics Committee, Faculty of Health Sciences, University of KwaZulu-Natal (Reference number BE098/18).

## Results

The themes and subthemes of the findings are presented here in narrative form, according to the constructs of the Health System Dynamics framework, starting with Service Delivery. Direct quotations are added to illustrate key points.

### Service delivery

Themes under this component will describe the structure of the CAMH system in the Amajuba District. This includes a general “Overview of CAMH services”, and “Identification and referral”.

#### Overview of CAMH services

CAMH services in Amajuba District Municipality were diverse. Public sector professional mental health services were provided in a largely centralised fashion by psychologists based at the district regional hospital. This hospital served as a referral point for at-risk learners identified within the school system. Service providers who helped to identify and refer children and adolescents potentially requiring mental health care were situated at different levels of the community, health and education systems, and included nurses in clinics, social workers in the communities, educators, learner support agents and school health nurses in schools. Beyond the public health system, there were also a variety of non-government service providers who provided mental health services such as awareness campaigns, assessment and referrals to a limited degree. This included general practitioners, religious counsellors, non-governmental/non-profit organizations (NGOs/NPOs) and traditional healers.

In terms of the content of CAMH services, health care involved psychotherapy and psychopharmacological support, largely provided in the hospitals. Educators and caregivers mentioned additional interventions to assist children in the school environment and at home. Extra classes were organized for learners identified to be dealing with psychological challenges and struggling academically. They expressed that these interventions were insufficient and were negotiating for professional psychological assistance for the learners from the Department of Education. Further, the Department of Social Development provided disability grants to children with intellectual disabilities and autism, illustrated by the following:

"I was advised to register her for the disability grant from the government, so that helps cater for her needs. We are fine financially because she receives the grant." (Caregiver 4).

#### Identification and referral

A service that was described as especially problematic was early identification of CAMH problems and appropriate referral; with most CAMH conditions identified and referred by the school system—but were generally quite late in the illness progression, when they were affecting children's academic performances. Very few cases were identified by health workers in hospitals, PHC clinics, Ward-based Primary Health Care Outreach Teams (WBPHCOTs), or by the caregivers. This finding was illustrated by the following:

"In most cases what I found is that children are identified by their educators. They are identified there in school and then referred to the clinic and then from the clinics to us here. And, there are few cases where children are brought to the Hospital for other things and mental health issues are picked up as a secondary problem that is seen, but otherwise in most cases it's the educators unless a child has a clear mental health issue that is visible then the child is brought into the health system by the caregiver." (Clinical Psychologist 1).

Once a child has been identified as needing mental health care, further steps depend on the specific space where identification occurred, and the nature of the perceived need. The educators and learning support agents (LSA) in schools mentioned that they provided some initial assessment and interventions before referring the children for further care. However, four of the twelve schools visited within the district still did not have any skilled staff or resources to provide initial CAMH assessment or interventions to assist their learners, they also did not have any information on the referral pathways. Integrated School Health Programme (ISHP) teams were yet to adopt mental healthcare into their activity portfolio.

"We identify learners who have special needs, behavioral problems or learners who are abused physically, emotionally and socially. Firstly, we screen those learners, fill the necessary forms and then we sit down with the learners to find out what the problem is, identify how we can help and if we cannot help, we call in supervisors from the DBE district office, then they will come and assist. They either do one-on-one sessions or sometimes they will take a group for assessment. After assessing them, if they see that the learners do have problems, they refer those learners to special schools. If it's a behavioral problem, they make sure that they do follow-up interventions like counselling or social work consultation and they refer some of the learners to the psychologists." (LSA, school C).

A principal mentioned the need to train educators to prevent inappropriate referral and labelling.

"….to take this matter seriously we need some resources to assist the schools, then the training of teachers also is important. I don't want teachers to wrongly identify and say it behaviour problem when the learner does not want to write due the relationships you have with that learner—so training of teachers is very important- so that they can be able to identify the learner." (Principal 1).

A senior mental health professional highlighted that the psychologists are mostly the first point of contact for children and adolescents with CAMH conditions within the hospital (most of the referrals from the schools are addressed to them) and they refer them to the appropriate specialists for cases in need of more specialized interventions. According to one of the psychologists:

"When they come to us, they are mostly accompanied by their caregivers, if maybe they come from school they come with their educators. So, we do the debriefing to sort of understand the child's condition and give us a picture of what is going on so that we can determine which services they need, and then if they need to be referred to other specialists, we do that. (Clinical Psychologist 1).

The psychologist also mentioned inappropriate referral from schools, children with learning disabilities that should be referred to educational psychologists are referred to the clinical psychologists. This is due to the shortage of educational psychologists in the district, thereby resulting in back referral*.*

"Children with learning difficulties are often referred to us but we always refer them back to the department of education because they have an educational psychologist. We understand that she is the only one for the district, and she's not coping. Because of this, schools tend to push them towards the department of health, but we don't do those assessments". (Clinical psychologist 1).

### Resources

The availability and organization of CAMH resources in the district are presented below, according to human resources, infrastructure, and supplies, knowledge, and information.

#### Human resources

Participants described a severe shortage of human resources to deal with CAMH problems within the Departments of Health and Basic Education. The service providers within DoH mentioned that they are overwhelmed due to limited CAMH human resources, increasing CAMH workload and inadequate CAMH training for non-specialists.

"…it’s tiring and frustrating. Because sometimes the available resources are inadequate…although, Madadeni hospital started child psychiatry training for professional nurses. The first Nurse was trained last year and two professional nurses are currently undergoing training at Bloemfontein. I think they are still not enough considering the number of cases we see” (Psychiatric nurse 1).

There was a widely-held view that CAMH services are limited in the district, but there was also sympathy from several participants that the few service providers were doing their best, and—under the circumstances—purportedly provided highly responsive care. Caregivers were appreciative of the good communication and friendly engagement of key mental health professionals. This was illustrated by the quotation below:

“We got a very great help, they really helped us, especially the Provincial hospital… the services were very good, and they were very helpful. The medication he receives here is helping a lot. They communicate with me properly, I was even able to ask questions and they could answer, they have been very caring towards me and the child, so I can say it was very good.” (Caregiver 2).

The lack of mental health human resources, and the resulting limitations in providing care, was bemoaned by one mental health participant as follows:

Unfortunately, we can’t see them more than once a month like everyone else because of staff shortage. However, if there is an urgent need for treatment, like sometimes we do fear that these persons might do something to harm themselves then we try to squeeze them in, but we just see them once a month. We usually make appointments in the mornings for people to come and see us… However, for school going-children we do make provisions for them, we see them in the afternoons, we schedule their appointments for 2 pm, so that at least they will be able to go to school in the morning.” (Psychologist 1).

Some medical professionals noted that CAMH services provided opportunities for self-development, as most of them are medically qualified professionals without formal qualifications in psychiatry or child and adolescent psychology.

“I enjoy providing CAMH services …it’s very interesting and challenging but I learn from the experience and it motivates me to develop my skills…I was working with a doctor who was about to retire so I joined her and she exposed me to one or two things before she left. I have some years of experience in it now, but I’m not a child and adolescent specialist, we don’t have any in the district as well.” (Medical officer 1).

The psychiatrist suggested that the CAMH system could be strengthened through the development of outreach teams to expand the CAMH workforce, ensure consistent in-service training across all the departments involved in delivering CAMH services, particularly for PHC nurses to facilitate the integration of CAMH services into primary health care, conduct awareness campaigns and provide psychosocial support to families to strengthen the existing CAMH system.

“I suggest the development of a Psychiatric Outreach Team consisting of professional Nurses, Staff Nurses and Social Workers. They need to go and visit Schools so that they can do in-service training and awareness campaigns… visit families because they need to capacitate them and support them. Also, training, I have been yearning for this, the PHC Staff members should undergo CAMH training.” (Psychiatrist).

#### Infrastructure and supplies

Findings revealed that there were very few special schools catering for children with special needs in the district, and only two of them were equipped to admit children with CAMH conditions. An educator from one of the two schools stated that the school was overpopulated due to the increasing prevalence of CAMH in the district:

“At first, we had the capacity of 150, but due to the increasing number of children with mental disabilities we have about 350 leaners, our school is full.” (Educator 2, special school 1).

There was widespread concern about the challenge of finding suitable schools for children whose mental health needs could not be met by their current schools. Some children were not enrolled into school at all, because they were rejected by the mainstream schools, with the limited special schools available in the district being overwhelmed due to the lack of space and shortage of resources. A caregiver relates this as follows:

“I once struggled to find a school for him and I am still having that challenge because I am yet to find one that can accept him.” (Caregiver 7).

In cases where caregivers were successful in placing their children in special schools, they received additional support in the form of transport services, as described below:

“He is now studying in a special school, where they have trained teachers who are knowledgeable about his condition, so I am happy he is in the right place. They taught him how to write when he got there…he's now trying to write his name. It is just okay because they also provide him with transport.” (Caregiver 13).

The chief director of special schools from the district Department of Education explained the school placement procedure.

“First, we do the placement assessment, when a leaner is referred for special school placement. A committee which consist of an occupational therapist, physiotherapist, the HOD and the class teacher will sit to decide. We assess the physical ability of the child and then cognitive assessment all these assessments will assist us with class placement. You know, sometimes the learner comes to us at the age of 10 and never accessed any form of education, but we can’t place them in the first year of School. After series of assessments, once we realize the level of assistance needed by the learner, we then recommend placement, we will then ask the parents to sign a consent form where they would agree that the learner should be enrolled into a special school.” (Chief director, special schools).

A caregiver also voiced her concern about the lack of higher education or opportunities for career development for adolescents with mental disabilities.

“My worry is that when they reach the age of 18 they should not just stay home, there must be something for them to do because people take advantage of children in these kinds of conditions because a lot of them tend to wonder in the street after they leave school. Maybe the government could help build a school that can take those that are over the age of 18.” (Caregiver 17).

#### Knowledge and information

There seemed to be a lack of knowledge in communities on identifying mental health symptoms at an early stage. In some cases, caregivers noticed some symptoms at an earlier stage, but they couldn’t specify the nature of condition and did not access care for the child until they were identified and referred from school. These caregivers also mentioned that they could not seek help for the children because they didn’t have a clear understanding of the conditions, where and how to seek medical care. This is illustrated below:

“I noticed before the school called me, but I couldn’t take any step because I didn’t know what the problem was and where to take him for treatment until he was referred by the school, they gave me a letter and I took her to the hospital.” (Caregiver 14).

Some caregivers reported that they noticed certain symptoms of abnormality. Although they couldn’t ascertain the nature of the problem, they immediately sought help for the child. Two of the caregivers took their children to the clinics close to them and were referred to the hospital while others took their children directly to the hospital. However, the caregivers who took their children directly to the hospital mentioned that they were requested to obtain referral letters from the school or a clinic. The following excerpt refers:

“We noticed the problem at home, but we couldn’t identify it as autism, so I brought him here to the hospital but then they said I should get a letter from his school about his condition.” (Caregiver 11).

### Population

The results under this component reveal the characteristics of the CAMH service users mainly caregivers of children with CAMH challenges in the district.

Government stakeholders described particular challenges in engaging with caregivers of children and adolescents with mental health needs. Many caregivers were yet to accept their children’s conditions and struggled to comply with the prescribed treatment regimen, and highlighted below:

“I love working with the children but some of the caregiver are in denial they don’t adhere to what you tell them whether its homework, time keeping, bookkeeping. It’s kind of frustrating because you know the child should be improving, but the child is not because the parent or caregivers are not adhering.” (Psychologist 1).

The challenging nature of child and adolescent mental health conditions led to many of the caregivers describing feelings of concern, helplessness and exhaustion, as expressed below:

“I cried a lot and even now I haven’t accepted it because I have two children, both have same condition. I accepted with the first one, but I couldn’t accept with the second one. It was really hard, and people were talking all they want about me and making fun of me that they rejected my children from school.” (Caregiver 4).

The complicated nature and under-resourcing of CAMH conditions further have a substantially negative effect on educators, not to mention the critical weight such conditions have on children’s functioning, daily interactions with their environment, emotions, behaviors and academic performance, resulting in, among others, poor academic performance, school truancy and drop-out. The below quotation refers:

“Their conditions affect us a lot; particularly it makes me sad. It affects us to such an extent that we end up not knowing what to do because we encounter such problems each and every day and there is no way we can help the children. It also affects their academic performance many of them are not doing very well academically, and some of them exhibit some behavioral problems. Sometimes we spend extra time to assist some of them, we visit their homes and even give some learners money to buy grocery.” (Educator 2).

### Leadership and governance

Participants pointed to the lack of a coordinated system of CAMH care as a major barrier to providing and accessing CAMH services in the district. This was exemplified by, particularly, poor intersectoral collaboration, and the lack of a standardised procedure and coordination for delivering CAMH services across the various departments in the district. There were no adequately integrated procedures for managing and reporting CAMH cases.

One participant referred to the overall system of care for children living with CAMH conditions in the district as “disjointed”. An example of this disjointedness was that certain services were packaged for children in different age groups across the two hospitals, which often required caretakers to find means of transporting the children between the hospitals to access different specialist services. This is illustrated in the quotation below:

“The system is a bit disjointed, for children below 12 years, we still have to refer them to Newcastle hospital for occupational therapy and speech therapy. It would’ve been better if we had everything in one place, but for children above 12 years, it’s better because everything is here.” (Occupational therapist hospital A).

### Context

Factors that were perceived to impede CAMH service provisioning from the wider contexts of the district emerged. The coalescence of the district disease burden and resource shortages resulted in very limited health awareness being conducted, which in turn resulted in poor mental health literacy. Tied to this barrier, it was often mentioned that there are high levels of stigma towards mental illness among children and adolescents, illustrated by the following:

“She does get discriminated which is something that pains me a lot. We are even afraid to send her to the shops and they even discriminate her because of the school she is going to.” (Caregiver 14).

Dysfunctional family systems were raised as a major risk factor and barrier to accessing CAMH services for children. The participants particularly emphasized the absence of parents—leaving children to the care of grandparents and other family members or leaving adolescents to care for themselves as a major problem in the community. The following quotation illustrates this point:

“…most are from broken families; they stay with elderly people and we’ve got children heading the family.” (Principal).

“Some of the parents are not staying with their children, they work and stay out of town… They come on month ends—just providing money—and leave the children to guide themselves. Some children are in distressful situations because they were in a way abandoned by their parents.” (SANCA coordinator).

## Discussion

The study sought to explore service providers and service users’ experiences of providing and accessing CAMH services and their perceptions of the available CAMH services in the district using the health system dynamics framework.

Key barriers and facilitators emerged for CAMH in the Amajuba District Municipality. Certain community factors such as low mental health literacy resulting in misconceptions and stigmatization, and the dysfunctional nature of the family system within the communities were highlighted as major CAMH risk factors within the district that impedes access to CAMH services. Community-based stigma can prevent caregivers from seeking help for their children, Heflinger and Hinshaw [23] stated that stigmatization increases the burden caused by mental illness and is a major barrier to accessing and utilizing mental health services. According to Brannan and Heflinger [7], caregivers of children with mental disorders often experience the pernicious impacts of stigma and therefore delay accessing mental health services for their children.

The study further revealed that the shortage of resources particularly CAMH specialists, lack of intersectoral collaboration and poor coordination, financial restrictions, and the low priority given to CAMH services in the district negatively impacts on the state of CAMH and serves as barriers to accessing CAMH services in the district. Nevertheless, the few available CAMH specialists were perceived to be competent and dedicated to delivering quality services but could benefit from systems strengthening initiatives that could expand the workforce and equip them with the required skills, resources and adequate coordination. These findings corroborate the findings of a recent study conducted in the Western Cape Province of South Africa by Mokitimi et al. [32] which highlighted inadequate CAMH resources, lack of priority for CAMH services and low levels of advocacy for CAMH services as major weaknesses of CAMH services in the province.

The shortage of educational psychologists which resulted in inappropriate referrals, disruption of assessment procedures for children with intellectual disabilities and increased workload for the limited available clinical psychologists was reported as a major barrier to CAMH services by the DoH stakeholders. Hence, the need to employ more educational psychologists by the Department of Education to address the needs of children with learning challenges was suggested. Stakeholders also suggested the provision of in-service CAMH training for psychiatric nurses, school health nurses, social workers and PHC workers which could facilitate the adoption of a task-sharing approach considering the shortage of CAMH specialists in the district.

While schools play a vital role in the identification and referral of CAMH challenges [36], the DBE stakeholders reported that they lack the required skills, time and tools to adequately screen and refer children thereby hindering many children and adolescents living with CAMH conditions from accessing the required CAMH services. The lack of appropriately defined referral pathways for children and adolescents identified as having mental health problems also emerged as a major barrier to providing adequate CAMH services within the school environment. As mentioned earlier, the majority of children within the school environment identified as in need of mental health services were referred directly to the hospitals which resulted in bottlenecks, with long waiting lists. Therefore, the DBE stakeholders suggested that efforts to build teachers’ capacity to facilitate early identification, screening and referral for children and adolescents at risk to optimize their health and development, as well as their academic potential, should be explored. This would assist the teachers to distinguish between learning problems that should be referred to educational psychologists, social problems that require social work interventions and mental health conditions that require the services of clinical/counselling psychologists. A study conducted by Cappella et al. [9], emphasized the significant roles of teachers in delivering CAMH services. They proposed the use of an ecological model to strengthen teachers’ capacity and facilitate active collaboration with mental health specialist for the reformation of school-based mental health services in low resource settings.

The study underlined the lack of a coordinated and integrated system of CAMH services particularly the lack of collaboration between the different sectors providing CAMH services in the district. This lack of adequate coordination and collaboration accounts for the inadequate communication between the different sectors, undefined screening/assessment procedure and referral pathways which results in delayed access to mental health care and the development of required interventions to address the various conditions affecting children. This finding is similar to the findings of previous studies conducted in Ghana, Uganda, Zambia and South Africa [27, 33, 43] which identified the consequences of a weak intersectoral collaboration for the delivery of mental health services particularly CAMH services in low resource settings.

The study participants emphasized the impact of CAMH conditions on the academic performance of children and adolescents which is further compounded by the shortage of special schools, the difficulties associated with securing school placements, the inadequate attention paid to the quality of education obtained and the lack of opportunities to pursue higher or vocational education after completing basic education for children and adolescents with CAMH challenges. Many children and adolescents living with learning disabilities are not receiving the required educational help for their special needs leaving them to helpless. This finding corroborates the findings of a study conducted in a South African peri-urban township by Saloojee et al. [41] who found that many children with intellectual disabilities are not enrolled in schools.

The caregivers mentioned financial constraints, lack of knowledge on how to access the available services and lack of psychosocial support which they encountered daily in their pursuit to alleviate the conditions of their children. Previous studies [2, 12, 21, 28, 34, 39] have also highlighted the psychological, physical and financial burden associated with caring for people with mental health challenges and the need to develop interventions that would equip caregivers with skills to alleviate these burdens.

Caregivers are central to CAMH prevention and effective management but require consistent support to acquire the necessary coping, communication, resilience, problem-solving and stress management skills. Moreover, the need for intensive CAMH awareness programs was suggested by the participants as well as the need to organize CAMH outreach teams to disseminate CAMH information and implement community based CAMH services in the district. According to the participants, these strategies will increase the knowledge of CAMH within the communities and could eliminate stigma and misconceptions around CAMH conditions. However, Hinshaw [24] proposed that stigma operates on multiple levels and mere public education programs might not resolve the problem of stigmatization. Therefore, the need to incorporates different change strategies targeted at the different interacting levels within the communities is required.

### Limitations

While a purposive sampling technique was used in selecting the study participants to obtain in-depth information on the current state of CAMH in the district, we acknowledge the various categories of stakeholders were a product of the differential availability of the stakeholders. It is possible that we might not have adequately captured the perspective of other key informants, particularly those within other sectors outside the DBE, DOH and NGOs/ CBOs partnering with DOH and DSD. However, the study included different categories of stakeholders to obtain rich data about the experiences and perceptions of CAMH service delivery in the district.

### Recommendations

The findings of this study suggest the need to create a district CAMH intersectoral coordinating or liaison forum to facilitate joint CAMH service planning and implementation to develop intersectoral agreements, developing defined referral pathways between relevant sectors, mobilizing resources, optimizing available resources within each sector, clarifying roles and responsibilities of the different sectors, promoting awareness and staff training on CAMH. Moreover, the need for continuous in-service training and capacity building through supervision and mentorship for stakeholders in each of the sectors cannot be overemphasized as in-service training, mentorship and specialists support can facilitate the acquisition and the willingness to implement new skills. Additionally, the development of management guidelines specifying the management procedures (identification, assessment, referral, treatment/interventions) for each sector and at the different levels of care should be prioritized.

It is important to address the educational needs of children and adolescents living with CAMH challenges by mobilizing resources such as providing learning equipment, building more classrooms and creating professional support teams to expand the capacity of the available special schools to accommodate children and adolescents living with severe CAMH conditions specifically learning difficulties in the district.

Increased attention should also be paid to educating and providing the necessary socioeconomic support for caregivers of children and adolescent with CAMH conditions. Caregivers should be sensitized about the importance of actively participating and complying with the management regimen recommended for their children’s conditions within the health care system and school. It is also important to invest in a rigorous approach to disseminating mental health education especially CAMH information within the district to eliminate discrimination and stigma. These information dissemination strategies should include the transmission of CAMH messages using public-social media platforms, ensure regular CAMH information contacts at the community levels and provide adequate support and education at the family level.

## Conclusions

In conclusion, the need to build the capacity of all the involved stakeholders in relation to CAMH services is imperative in the district. Although teachers and caregivers are not in a position to treat CAMH conditions, they can be equipped to identify children and adolescents with incipient mental health problems so that they access care early on in the illness progressions. They can also be equipped with knowledge and skills to support children and adolescents with mental health problems and adhere to management regimens. Teachers could be assisted to promote mental health and resilience, identify and refer CAMH conditions through enhancing their mental health literacy and providing them with validated and appropriate screening tools. Creating mental health outreach teams could further facilitate CAMH awareness within the communities thereby enhancing CAMH literacy and access to quality CAMH services. This could also potentially relieve the burden of care placed on the limited specialists and ensure a functional and sustainable collaborative system of CAMH care in the district.

## Data Availability

The datasets used and analysed during the current study are available from the corresponding author on reasonable request.

## Abbreviations

CAMH: Child and adolescent mental health
DoH: Department of Health
DBE: Department of Basic Education
DSD: Department of social welfare
LSA: Learner support agent
NGO: Non-governmental organizations
NPO: Non-profit organizations
PHC: Primary Health Care
SANCA: South African National Council on Alcoholism and Drug Dependence

## References

[1] Amajuba district municipality spatial development framework. (SDF). Amajuba District Municipality. https://www.amajuba.gov.za/media/1165/annexure-a-sdf.pdf (2017/2018).

[2] Ambikile JS, Outwater A (2012). Challenges of caring for children with mental disorders: experiences and views of caregivers attending the out-patient clinic at Muhimbili National Hospital, Dar es Salaam-Tanzania. Child Adolesc Psychiatry Ment Health.

[3] Analytics G. Evaluation of phase 1 implementation of interventions in the National Health Insurance (NHI) pilot districts in South Africa. Johannesburg: Genesis Analytics. 2019. https://www.hst.org.za/publications/NonHST%20Publications/nhi_evaluation_report_final_14%2007%202019.pdf.

[4] Babbie E, Mouton J (2001). The practices of social research (South Africa edition).

[5] Babatunde GB, van Rensburg AJ, Bhana A, Petersen I (2019). Barriers and facilitators to child and adolescent mental health services in low-and-middle-income countries: a scoping review. Glob Soc Welf.

[6] Babatunde GB, Bhana A, Petersen I (2020). Planning for child and adolescent mental health interventions in a rural district of South Africa: a situational analysis. J Child Adolesc Ment Health.

[7] Brannan AM, Heflinger CA (2006). Caregiver, child, family, and service system contributors to caregiver strain in two child mental health service systems. J Behav Health Serv Res.

[8] Braun V, Clarke V (2006). Using thematic analysis in psychology. Qual Res Psychol.

[9] Cappella E, Frazier SL, Atkins MS, Schoenwald SK, Glisson C (2008). Enhancing schools’ capacity to support children in poverty: an ecological model of school-based mental health services. Adm Policy Ment Health Ment Health Serv Res.

[10] Collins PY, Insel TR, Chockalingam A, Daar A, Maddox YT (2013). Grand challenges in global mental health: integration in research, policy, and practice. PLoS Med.

[11] Darlington Y, Feeney JA (2008). Collaboration between mental health and child protection services: professionals' perceptions of best practice. Child Youth Serv Rev.

[12] Datta SS, Russell PSS, Gopalakrishna SC (2002). Burden among the caregivers of children with intellectual disability: associations and risk factors. J Learn Disabil.

[13] De Vos AS, Strydom H, Fouche CB, Delport C (2002). Research at grass roots.

[14] Department of Health (2003). National policy guidelines for child and adolescent mental health, 2003.

[15] Department of Health (2011). National health insurance in South Africa: policy paper. Gov Gaz.

[16] Department of Health (2013). National mental health policy framework and strategic plan 2013–2020.

[17] Erskine HE, Baxter AJ, Patton G, Moffitt TE, Patel V, Whiteford HA, Scott JG (2017). The global coverage of prevalence data for mental disorders in children and adolescents. Epidemiol Psychiatr Sci.

[18] Gale NK, Heath G, Cameron E, Rashid S, Redwood S (2013). Using the framework method for the analysis of qualitative data in multi-disciplinary health research. BMC Med Res Methodol.

[19] Golberstein E, Wen H, Miller BF (2019). Coronavirus disease 2019 (COVID-19) and mental health for children and adolescents. JAMA Pediatr.

[20] Govender K, Penning S, George G, Quinlan T (2012). Weighing up the burden of care on caregivers of orphan children: the Amajuba District Child Health and Wellbeing Project. South Africa AIDS Care.

[21] Green SE (2007). “We’re tired, not sad”: benefits and burdens of mothering a child with a disability. Soc Sci Med.

[22] Haine-Schlagel R, Mechammil M, Brookman-Frazee L (2017). Stakeholder perspectives on a toolkit to enhance caregiver participation in community-based child mental health services. Psychol Serv.

[23] Heflinger CA, Hinshaw SP (2010). Stigma in child and adolescent mental health services research: understanding professional and institutional stigmatization of youth with mental health problems and their families. Adm Policy Ment Health Ment Health Serv Res.

[24] Hinshaw SP (2005). The stigmatization of mental illness in children and parents: developmental issues, family concerns, and research needs. J Child Psychol Psychiatry.

[25] Juengsiragulwit D (2015). Opportunities and obstacles in child and adolescent mental health services iFn low-and middle-income countries: a review of the literature. WHO South-East Asia J Public Health.

[26] Klasen H, Crombag AC (2013). What works where? A systematic review of child and adolescent mental health interventions for low- and middle-income countries. Soc Psychiatry Psychiatr Epidemiol.

[27] Kleintjes S, Lund C, Flisher AJ (2010). A situational analysis of child and adolescent mental health services in Ghana, Uganda, South Africa and Zambia. Afr J Psychiatry.

[28] Kuo DZ, Cohen E, Agrawal R, Berry JG, Casey PH (2011). A national profile of caregiver challenges among more medically complex children with special health care needs. Arch Pediatr Adolesc Med.

[29] Kutcher S, McLuckie A (2009). Evergreen: towards a child and youth mental health framework for Canada. J Can Acad Child Adolesc Psychiatry.

[30] Macdonald W, Bradley S, Bower P, Kramer T, Sibbald B, Garralda E, Harrington R (2004). Primary mental health workers in child and adolescent mental health services. J Adv Nurs.

[31] Matsoso MP, Fryatt R (2013). National Health Insurance: the first 18 months. SAMJ.

[32] Mokitimi S, Jonas K, Schneider M, De Vries PJ (2019). Child and adolescent mental health services in South Africa—senior stakeholder perceptions of strengths, weaknesses, opportunities and threats in the Western Cape Province. Front Psychiatry.

[33] Mokitimi S, Schneider M, de Vries PJ (2018). Child and adolescent mental health policy in South Africa: history, current policy development and implementation, and policy analysis. Int J Ment Health Syst.

[34] Murphy NA, Christian B, Caplin DA, Young PC (2007). The health of caregivers for children with disabilities: caregiver perspectives. Child Care Health Dev.

[35] O’Brien D, Harvey K, Howse J, Reardon T, Creswell C (2016). Barriers to managing child and adolescent mental health problems: a systematic review of primary care practitioners’ perceptions. Br J Gen Pract.

[36] O’Reilly M, Adams S, Whiteman N, Hughes J, Reilly P, Dogra N (2018). Whose responsibility is adolescent’s mental health in the UK? Perspectives of key stakeholders. Sch Ment Health.

[37] Paruk S, Karim E (2016). Update on adolescent mental health. SAMJ.

[38] Polkinghorne DE (2005). Language and meaning: data collection in qualitative research. J Couns Psychol.

[39] Rammohan A, Rao K, Subbakrishna DK (2002). Burden and coping in caregivers of persons with schizophrenia. Indian J Psychiatry.

[40] Ritchie J, Lewis J, Nicholls CM, Ormston R (2013). Qualitative research practice: a guide for social science students and researchers.

[41] Saloojee G, Phohole M, Saloojee H, IJsselmuiden C (2007). Unmet health, welfare and educational needs of disabled children in an impoverished South African peri-urban township. Child Care Health Dev.

[42] Simons H (2009). Case study research in practice.

[43] Skeen S, Kleintjes S, Lund C, Petersen I, Bhana A, Flisher AJ, The Mental Health and Poverty Research Programme Consortium (2010). ‘Mental health is everybody's business’: roles for an intersectoral approach in South Africa. Int Rev Psychiatry..

[44] Smith J, Firth J (2011). Qualitative data analysis: the framework approach. Nurse Res.

[45] Staniford LJ, Breckon JD, Copeland RJ, Hutchison A (2011). Key stakeholders’ perspectives towards childhood obesity treatment: a qualitative study. J Child Health Care.

[46] Van Olmen J, Crie B, Bhojani U, Marchal B, Van Belle S, Chenge F, Hoeree T, Pirard M, Van Damme W, Kegels G (2012). The health system dynamics framework: the introduction of an analytical model for health system analysis and its application to two case-studies. Health Cult Soc.

[47] Vandenbroeck P, Dechenne R, Becher K, Eyssen M, Van den Heede K (2013). Soliciting stakeholders’ views on the organization of child and adolescent mental health services: a system in trouble. Child Adolesc Psychiatry Ment Health.

[48] Watson R, McKenna H, Cowman S, Keady J (2008). Nursing research: designs and methods e-book.

[49] World Health Organization (2005). Child and adolescent mental health policies and plans.

[50] World Health Organization (2008). Integrating mental health into primary care: a global perspective.

[51] World Health Organization (2008). mhGAP: Mental Health Gap Action Programme: scaling up care for mental, neurological and substance use disorders.

